# A non-lethal method to assess element content in the endangered *Pinna nobilis*

**DOI:** 10.1038/s41598-021-98535-2

**Published:** 2021-09-28

**Authors:** Devis Montroni, Andrea Simoni, Viviana Pasquini, Enrico Dinelli, Claudio Ciavatta, Carla Triunfo, Marco Secci, Claudio Marzadori, Pierantonio Addis, Giuseppe Falini

**Affiliations:** 1 Dipartimento di Chimica “G. Ciamician”, Alma Mater Studiorum − Università di Bologna, via F. Selmi 2, 40126 Bologna, Italy; 2 DiSTA, Department of Science and Technology of Agriculture and Environment, Alma Mater Studiorum - Università di Bologna, via Fanin 40, 40127 Bologna, Italy; 3 Dipartimento di Scienze della Vita e dell’Ambiente, Università di Cagliari, via Fiorelli 1, 09126 Cagliari, Italy; 4 Dipartimento di Scienze Biologiche, Geologiche e Ambientali, Alma Mater Studiorum − Università di Bologna, piazza di Porta San Donato 1, 40126 Bologna, Italy; 5 Istituto per lo Studio dei Materiali Nanostrutturati (CNR-ISMN), Consiglio Nazionale delle Ricerche, Via P. Gobetti 101, 40129 Bologna, Italy; 6 Fano Marine Center, The Inter-Institute Center for Research on Marine Biodiversity, Resources and Biotechnologies, Viale Adriatico 1/N, 61032 Fano, Italy; 7 Present Address: Department of Materials Science and Engineering, University of California, Irvine, CA 92697 USA

**Keywords:** Environmental chemistry, Environmental monitoring, Analytical chemistry

## Abstract

The fan shell *Pinna nobilis* is the largest bivalve endemic to the Mediterranean and is actually a strongly endangered species. Due to the biological, ecological, and historical relevance of this species, the research of a non-lethal method to relate the element content in organism’s tissues and environment can provide information potentially useful to evaluate environmental pollution and organism physiological status. In this study, a screening on element concentration in the animal growing environment (seawater and sediments) and in four soft tissues (hepatopancreas, gills, mantle, and muscle), and two acellular tissues (calcite shell layer, and byssus) was performed. The comparison among these results was used to assess whether the no-lethal acellular tissue element concentration can be used to reveal the element presence in the environment and soft tissues. Elements, such as B, Ag, As, Mn, Mo, Pb, or Se, showed a possible relationship between their presence in the byssus and soft tissues. In the byssus Cr, Sb, Sn, and V have shown to be mostly related to the environment, more than the soft tissues, and might be used to draw a historical record of the exposure of the organism. The element concentration in the calcite shell layer did not relate with environmental element concentrations. Essential elements, like Cu, Fe, Ni, and Zn, were present in calcite shell layer and byssus and are likely related to their biological activity in the organism. The research also gave an overview on the presence of pollution and on the preferential intake route of the element. In summary, this study, performed on a limited number of specimens of this protected species, indicated that element concentration in the byssus can be applied as non-lethal method to monitor this endangered species and its interaction with the elements in the growing environment.

## Introduction

**Figure 1:**
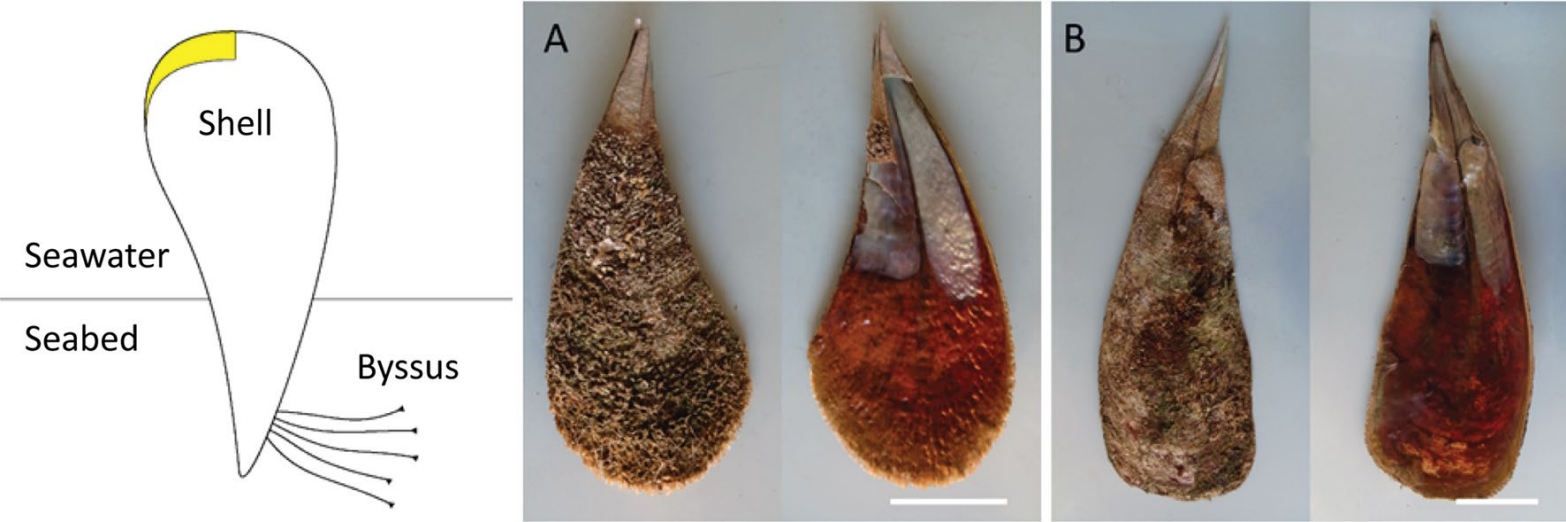
*P. nobilis* shells. (left) A schematic representation of a *P. nobilis*, in yellow the portion of shell collected for a first analysis. (right) Pictures of the extern and intern of the shell valves of *P. nobilis* from site (**A**) (about 40 cm long) and site (**B**) (about 60 cm long); two different morphology of the shell can be observed. Scale bar: 10 cm.

The fan mussel, *Pinna nobilis* (Linnaeus, 1758), is the largest endemic bivalve of the Mediterranean Sea and with its size (up to 120 cm in height) ranks as one of the largest worldwide^1,2^. Besides these aspects *P. nobilis* has also ecological, historical, and social relevance^3–5^. This species grows on soft-bottom coastal areas and mainly inhabits seagrass meadows (*Posidonia oceanica* and *Cymodocea nodosa*), but also occasionally thrives on unvegetated bottoms or among boulders^5–10^. *P. nobilis* has wide variation in shell size and its posture involves two-thirds of the shell projected into the water column (Fig. 1). It has been reported that smaller specimens would be more influenced by sediment and detritus pollution while larger animals would be more affected by the water column^11^. The large-sized *P. nobilis* ensure a wider contact surface with the external environment and a greater pollutant uptake than other mussels or clams^12^. In favorable conditions, the lifespan of *P. nobilis* is up to 50 years and this longevity ensures a cumulative uptake from the environment^13^. For example, a high amount of polyaromatic hydrocarbons was found in *P. nobilis* in the Balearic Archipelago a year after the oil spill accident^14,15^.

*P. nobilis* was a common inhabitant of Mediterranean coastal waters, but over the last few decades its population has drastically declined. This especially occurred in the last four years due to a mass mortality event. *P. nobilis* is now considered critically endangered (IUCN)^12,16,17^, and a protected species^18^. Therefore any new information on how anthropogenic activities affect its features is crucial for its preservation and recovery plans^16,19^.

The uptake of elements in *P. nobilis* has been explored only in few studies^15,20–24^. In 2014 Jebali et al.^24^, reported the irreversible presence of Cd, Fe, Mn, Pb, and Zn in gills and hepatopancreas in organisms transplanted first in a polluted environment and then in a protected unpolluted area. Vázquez-Luis et al.^15^, observed from 4 to 30 times higher metal content in *P. nobilis* compared to other bivalves, demonstrating interspecies variation in bioaccumulation between the different mussel genera.

Effects of contamination on the aquatic organism parameters have been investigated and employed as biomarkers in environmental quality assessment for years^25^. Bivalves assimilate pollutants by ingestion of particulate materials suspended in water or food, ion uptake of dissolved metals across gills, and adsorption on exposed tissue surfaces^26^. In fact, bivalves are commonly used to detect pollutants in the marine environment^27–34^, and, more recently, to assess changes in the status of the marine ecosystem in response to climate change^35,36^. Due to their wide distribution, sedentary state, ease of collection, and ability to accumulate high concentrations of a wide range of contaminants, including metals, bivalves were added in systematic monitoring in the early 70 s (i.e. the “Mussel-watch”)^24,33,37^. The association among traditional biochemical biomarkers with biometric, morphometric, and elemental composition of shells of calcifying organisms has been evaluated in several studies^25^. The changes observed in the elemental composition of shells suggested that exposure to contaminated environments can induce the biometric and morphometric alterations detected. However, the latter were influenced also by natural environmental conditions^38^. Compositional analyses also showed that organisms collected in more contaminated sites presented shells more fragile, with a reduction of thickness in the prismatic layer associated with the enlargement of the periostracum layer. In addition, a significant reduction in the intra-shell organic fraction amounts was observed in shells of organisms from the most contaminated site^39^.

Overall, the studies of bivalves’ bioaccumulation by metal pollution involve the dissection of specimens to collect soft tissues. So far, only a few studies considered both shells and soft tissues collection to correlate the metal presence^40,41^. Such approach is similar for humans, where sampling hairs or nails are generally considered for the analyses^42,43^. Moreover, shells have been used to determine the age or seawater temperature by studying the presence of isotopes or trace metals^1,12^.

This study aims to perform a wide screening to elucidate the distribution of different elements in diverse tissues of *P. nobilis* grown around the island of Sant’Antioco (Sardinia, Western Mediterranean, Italy). The element concentrations detected in tissues, combined with the quantification of these selected elements in the surrounding environment (seawater and seabed sediment), can indeed provide information on the organism response to their exposure. Most interestingly, the investigation on specific element concentration in two acellular matrices, the calcite shell layer and the byssus, and their correlation with those in soft tissues, might allow a monitoring tool for the environment and the organism without damaging or sacrificing the animal.

## Materials and methods

### Study area and sampling

**Figure 2:**
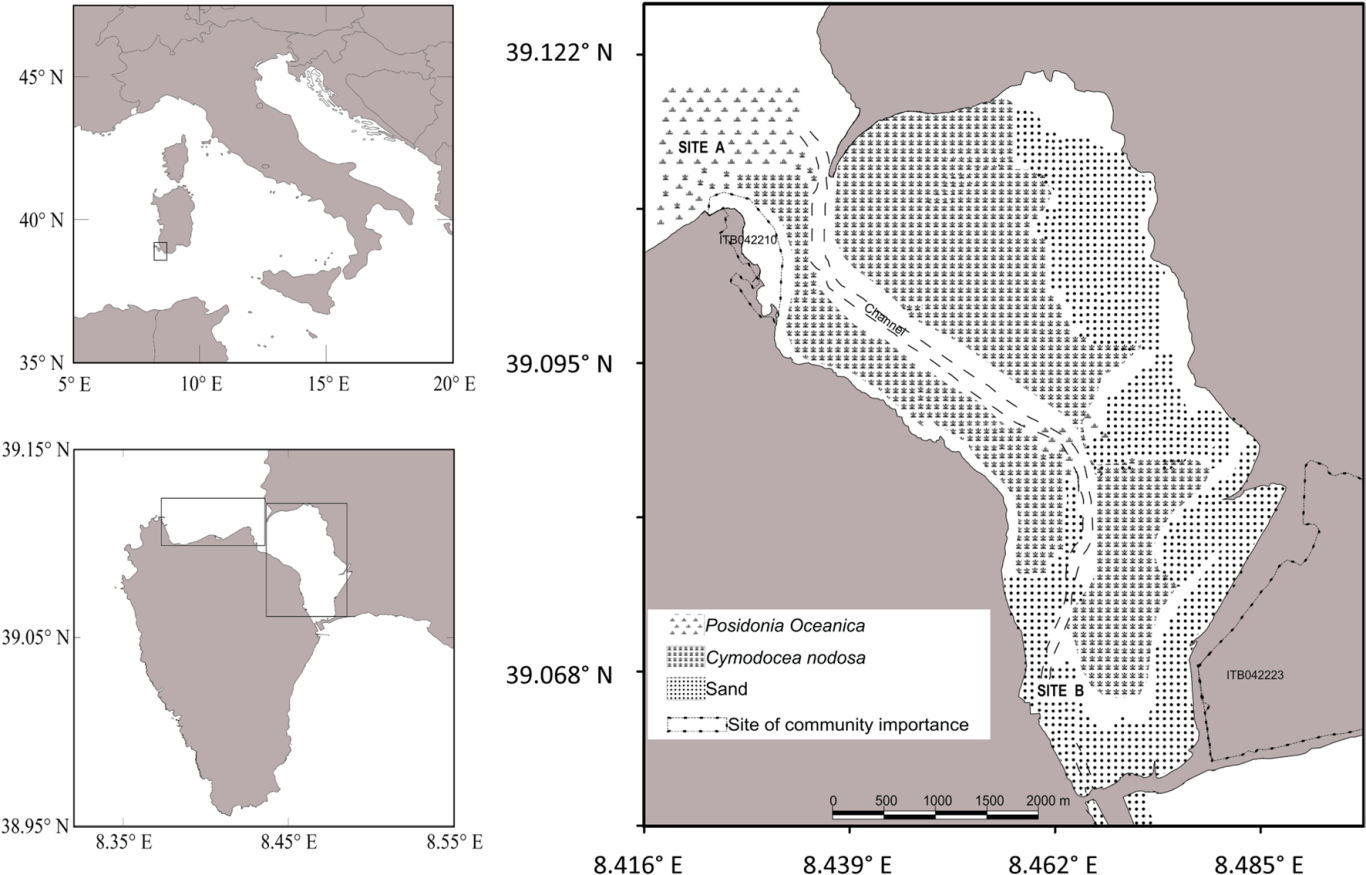
Map of the sampling sites in Sant’Antioco, Sardinia, Italy. The map on the right shows the lagoon of Sant’Antioco and the distribution of different habitats in it (from: Secci et al. modified)^6^.

The study was conducted in June 2017 around the Sant’Antioco Island (Sardinia, Western Mediterranean, Italy) prior to the mass mortality that affected the Mediterranean population size of *P. nobilis*. Two adjoining zones were considered for samplings: Sant’Antioco North (Site A) and Sant’Antioco South (Site B) (Fig. 2). Site A is an open sheltered bay (max depth = 2 m) that is exposed to NW winds. It is characterized by sandy, gently sloping bottoms, and is occupied by continuous and patchy meadows of *P. oceanica*, which provide a suitable habitat for the fan mussel. Site B is a shallow coastal lagoon (max depth = 1.5 m) located between the gently sloping coast of the island of Sant’Antioco and the mainland of Sardinia. It is connected to the open sea by one large inlet (1 km) in the north and a narrow inlet (60 m) in the south. The bottoms are characterized by mud and sand, and are occupied by mixed meadows of *P. oceanica* and *C. nodosa* interspersed with the green algae *Caulerpa prolifera*^6^.

This study has been conducted in full accordance with institutional, national, and international guidelines concerning the use of animals in research and/or the sampling of endangered species. A total of six specimens were collected by SCUBA diving under the approval of the Ministry for Environment, Land and Sea Protection of Italy [Permission in notwithstanding the provisions of the Decree DPR 357/97]. Two samples (30–40 cm long) were collected in site A, in a sandy seabed with *P. oceanica* meadows, and four (60–70 cm long) were collected in site B, a muddy seabed, taking the biggest specimen found from each site. The age of the animals was defined by counting the annual bands on the internal aragonitic posterior adductor-muscle scar (Fig. S1)^44,45^. We considered the shell muscle-scar rings developed annually in spring or early summer (when water temperature increase). Moreover, since in small Pinnidae (< 25 cm) the shell grows fast during the first year, we considered the first annual scar appearing in the second year. No spawning condition or sex of the *P. nobilis* samples were determined.

In each site, two seawater samples were collected at the depth of the first two organisms collected using a 50 mL cleaned polystyrene plastic flask. The samples were then filtered on a 0.25 μm syringe filter before the analysis. Sediment samples were collected within 2 m from the same specimen removing 1–2 cm of surface sediment and collecting the deeper sediment underneath in a 50 mL polystyrene plastic flask. Seawater and sediment samples were stored frozen. All the acids used in this study were trace element analysis grade. All solvents used to clean the matrices were at least HPLC grade.

### Sediment analysis

Three samples of sediment were collected from each site and stored in cleaned polystyrene containers. The frozen sediment samples were lyophilized leaving about 20–30 mL of sediment mixed with sea salt. An amount of 40 mL of Pre-milliQ water was added to each sample, which was then centrifuged at 1000×*g* for 5 min. The sediment was isolated and the process was repeated two more times. Then the sediment samples were lyophilized again and stored dried in a desiccator. These samples were homogenized and powdered with an agate mill. Pressed powder pellets were prepared for the determination of major and trace elements (Al_2_O_3_, CaO, Fe_2_O_3_, K_2_O, MgO, MnO, Na_2_O, P_2_O_5_, SiO_2_, TiO_2_, Ba, Ce, Cr, Co, Cu, La, Nb, Ni, Pb, Rb, S, Sr, V, Y, Zn, Zr) by means of X-ray fluorescence (XRF) spectrometry using a Panalytical Axios4000 automated spectrometer. Thermogravimetric analysis (TGA) was performed to quantify volatile compounds and carbonate presence. The system was pre-equilibrated at 30 °C, then a ramp from 30 to 1150 °C with a 12 °C min^−1^ heating rate was performed under nitrogen flow. The measure was performed at least two times on 10 mg of sample each time. The measure was performed using a SDT Q900 instrument (TA Instruments). More details concerning the analysis parameters for each sample and elements are reported in Tables S3 and S4.

Since Ag, B, Be, Cd, Li, Sb, Se, and Tl are not detectable using XRF, the sediments were analyzed for these specific elements using the ISO method 3052 “Microwave assisted acid digestion of siliceous and organically based matrices”. Briefly, up to 0.5 g of sediment were digested in 9 mL of concentrated nitric acid and 3 mL hydrofluoric acid for 15 min using microwave heating. Microwave digestion was performed using an Ethos Up Milestone digestion system. Samples were analyzed using an ICAP7000 Thermo ICP-OES. Fe and Zn gave no significantly different results using this analysis and XRF.

### Shell’s sample preparation

The shells (external tissue) were collected from the specimens and washed with abundant distilled water. Algae and other sessile organisms growing on the extern of the shell were removed manually or with a scalpel. To collect the most recent shell about 1 cm^2^ of the most external shell was removed using a saw with a diamond blade following the growing lines of the shell, as reported in Fig. 1. The shell sampling at the different annual growth was performed by cutting 1 cm^2^ at a distances in accordance with the growth curves reported in Richardson et al.^12^. The material collected was soaked in water and stirred for three hours, changing the water every hour, and cleaned using a brush to eliminate the mud residues. Once the shell was clean the organic matrix was removed by sodium hypochlorite treatment, soaking it in 50 mL of a 5 wt% solution for 1 h. Then the shell was collected and soaked in 50 mL of clean hypochlorite solution for 48 h. Once the treatment was completed the powder (composed of calcite pillars) obtained was filtered on a 0.2 μm filter, washed eight times with abundant milliQ water, and dried in a desiccator. About 1 g of pillar powder was set in 3 mL of milliQ water and dissolved adding 2.7 mL of HCl:HNO_3_ 3:1 slowly until complete dissolution of all the samples. The solution was then filtered on a 0.45 μm syringe filter and diluted to 10 mL using milliQ water. Syringe and filter were washed flushing four times 1 mL of milliQ water, which was collected in the final 10 mL of the sample. A control sample was obtained using the same amount of acid mixture and water. Prior to analysis the samples were conserved in closed plastic vials at room temperature.

### Tissues pre-treatment

The soft tissues analyzed were the digestive gland (also called hepatopancreas), the gills, the mantle, and the posterior adductor muscle. Once the tissue was collected by dissection, it was cleaned with abundant milliQ water, frozen with liquid nitrogen, and lyophilized. The dry tissue was weighted and conserved dry at 4 °C.

### Byssus pre-treatment

The byssus (external tissue) was collected by hand from the mollusk, rinsed with water to remove sand and mud, and dried in a desiccator. The bigger pieces of sediment, algae, or other materials glued to the plaques or entangled in the byssus were removed using a comb with close packed rigid teeth. Afterward, the byssus was washed with de-ionized water four times for 1 h, stirred twice in ethanol for 30 min, and washed again twice with Pre-milliQ water for 15 min. The clean byssus was conserved dry in a desiccator under vacuum.

### Organic matrices digestion

The elements inside the byssus and the tissues studied were quantified by digesting the sample. The sample was set in a Teflon holder with 0.5 mL of H_2_O_2_ (30 vol% Carlo Erba, for electronic applications) and 6 mL of nitric acid (65 vol% Honeywell). The holder was set in a microwave oven, Milestone, programmed to operate as follows: 2 min at 250 Watt, 2 min at 400 Watt, 1 min at 0 Watt, and 3 min at 750 Watt. The digested sample was quantitatively collected, diluted to 10 mL with water, and filtered on paper. This analytic procedure, along with the successive analysis using ICP-OES, was verified using a certified reference material (*Lagarosiphon major*, CRM 60; Community Bureau of Reference, Commission of the European Communities).

### Inductively coupled plasma–atomic emission spectroscopy (ICP-OES)

All the liquid samples obtained (seawater, acid-solubilized shells, and digested byssus and tissues) were measured three times, 12 s each, with 60 s of pre-running, using an ICP-OES, Spectro Arcos-Ametek, Inductive Coupled Plasma Optical Emission Spectroscopy with an axial torch and high salinity kit. The calibrating curve was made using certified standards in water.

More details concerning the analysis parameters for each sample and element are reported in Tables S2 and S3.

## Results

In this study, *P. nobilis* samples were collected inside (site A) and the other outside the coastal lagoon (site B) of Sant’Antioco (Sardinia, Italy). The sites differ for the geographical location, environmental conditions and the maximum length reached by individuals from the sampled population, about 40 cm and 65 cm for specimens from site A and B, respectively. The biggest organisms were collected from each population since those organisms have been exposed for the longest time to the environment. The age of the animals, reported in Table S1, was evaluated by counting the annual bands on the aragonitic posterior adductor-muscle scar in the shell, as: Age = (No of scars) + 1^1,12^. They ranges between 3 and 10 years old. In each site, seawater did not show common contaminants and the element concentrations were significantly similar (T-test, p = 0.05, ν = 2) between the two sites (Table S4), except for P (site A: 0.12 ± 0.02 μg g^−1^; site B: 0.05 ± 0.02 μg g^−1^). The sediment composition of the two environments was also observed to be significantly equal (T-test, p = 0.05, ν = 2). Despite this similarity (Table S4), site A appeared to be mostly Ca-based (site A: 35 wt%; site B: 12 wt%) while site B was mostly Si-based (site A: 46 wt%; site B: 69 wt%).

The shells (Fig. S2) are mainly composed of two mineralized layers. The first one, made of calcite pillars, composes most of the shell. The second one is the stiffer nacreous layer that covers the intern of about one-third of the shell (where most of the organism is located) and is used to anchor the adductor muscles. The element content of the calcite layer study was evaluated. The choice to use only the calcite layer, removing the external organic matrices (i.e. the periostracum), can appear counterintuitive since this acellular tissue was reported to host elements from the environment^46^. However, the periostracum, being the outermost region of the shell, is more subject to degradation and cross-contamination than the underlying mineralizing regions, especially by epibionts. For this reason, and in accordance with some literature (e.g.^47^) the periostracum was removed by the shells.

In the comparative studies among *P. nobilis* tissues only the youngest part of the shell was taken into exam, as shown in Fig. 1. The analysis showed (Table S4), as for the previous cases, almost no differences between sites A and B (T-test, p = 0.05, ν = 4). The only difference was found in the K concentration (site A: 330 ± 50 μg g^−1^; site B: 210 ± 20 μg g^−1^). In parallel, an investigation on the element content of the calcite shell layer was performed on all the shells sampling in correspondence of successive annual increments in the calcite shell layer. The data (Tables S5, S6) did not show any significant change among animals of different ages.

The second acellular matrix examined was the byssus, a matrix composed of proteic threads attached to particles of the substrate^48–50^. As before, only few metals have been observed being different between site A and B (T-test, p = 0.05, ν = 4): Ag (site A: not detected, < 0.04 μg g^−1^; site B: 0.25 ± 0.09 μg g^−1^), and Cr (site A: not detected, < 0.1 μg g^−1^; site B: 1.8 ± 0.7 μg g^−1^) (Table S4). This matrix generally showed a higher diversity in its element content, with Sb and Sn only detected in the byssus. As observed above also the soft tissues analyzed did not show significant differences in the element content between site A and site B (T-test, p = 0.05, ν = 4) (Table S7). The concentrations of typical physiological elements, Ca, K, Mg, and P, are reported in Table S8.

Since the two sites of sampling gave significantly similar results for both environment, tissues and external matrices, the two datasets were merged. This new dataset had a wider diversification of age from 3 to 10. However, such merging is also justified by the results on the investigations on the entire shells which show no significant change in elemental composition. The results are reported in Table 1.

**Table 1:** Average element content in the sample collected (A and B considered as a unique set).

	Seawater (μg g^−1^)	Sediment (μg g^−1^)	Shell (μg g^−1^)	Byssus (μg g^−1^)	Hepatopancreas (μg g^−1^)	Gills (μg g^−1^)	Mantle (μg g^−1^)	Muscle (μg g^−1^)
Ag	n.d.	n.d	n.d	**0.2 ± 0.1**	0.2 ± 0.2	1.1 ± 0.9	1.3 ± 0.5	0.2 ± 0.2
Al^a^	n.d.	40,000 ± 10,000	n.d	440 ± 80	60 ± 50	100 ± 100	200 ± 100	20 ± 10
As	n.d.	7 ± 2	n.d	2 ± 2	50 ± 10	30 ± 10	70 ± 30	20 ± 5
B	3.6 ± 0.1	10 ± 12	1.2 ± 0.2	200 ± 200	34 ± 9	16 ± 8	19 ± 6	17 ± 3
Ba*	0.025 ± 0.002	400 ± 200	1.1 ± 0.2	5 ± 2	5 ± 1	5 ± 6	3 ± 1	1.2 ± 0.3
Be	n.d.	n.d.	n.d.	n.d.	0.2 ± 0.08	0.04 ± 0.02	0.05 ± 0.01	0.023 ± 0.003
Cd	n.d.	n.d.	0.003 ± 0.004	0.08 ± 0.09	4 ± 4	7 ± 5	7 ± 8	4 ± 3
Co	n.d.	0.5 ± 0.9	0.2 ± 0.4	0.1 ± 0.2	20 ± 30	1 ± 2	0.3 ± 0.4	0.1 ± 0.2
Cr	n.d.	26 ± 5	0.6 ± 0.5	**1 ± 1**	n.d.	0.1 ± 0.2	0.4 ± 0.3	n.d.
Cu	n.d.	3 ± 3	0.08 ± 0.04	80 ± 30	19 ± 9	10 ± 10	11 ± 6	5 ± 2
Fe^a^	n.d.	6000 ± 3000	8 ± 3	1100 ± 300	330 ± 70	190 ± 80	200 ± 100	60 ± 10
Li	0.3 ± 0.01	n.d	2.5 ± 0.2	0.2 ± 0.1	1.7 ± 0.5	0.6 ± 0.3	1 ± 0.4	0.74 ± 0.1
Mn^a^	0.0118 ± 0.0008	140 ± 30	1.8 ± 0.6	1.2 ± 1	300 ± 100	400 ± 200	200 ± 100	140 ± 70
Mo	n.d.	4 ± 2	n.d	20 ± 20	n.d.	0.4 ± 0.6	0.3 ± 0.4	n.d.
Ni	n.d.	8 ± 2	0.3 ± 0.2	8 ± 6	3 ± 2	3 ± 2	2 ± 2	1 ± 1
Pb	n.d.	36 ± 10	2.2 ± 0.3	50 ± 20	7 ± 5	13 ± 6	12 ± 4	4 ± 4
S	890 ± 30	5,000 ± 3,000	2,200 ± 100	13,000 ± 1000	18,000 ± 1000	9000 ± 3000	10,000 ± 2000	9200 ± 600
Sb	n.d.	n.d.	n.d.	4 ± 5	n.d.	n.d.	n.d.	n.d.
Se	n.d.	5 ± 3	0.07 ± 0.05	5 ± 3	9 ± 2	6 ± 2	7 ± 2	2.6 ± 0.6
Si^a^*	–	220,000 ± 100,000	0.3 ± 0.2	40 ± 20	50 ± 20	19 ± 4	22 ± 5	13 ± 5
Sn	0.01 ± 0.01	3 ± 3	n.d.	1.7 ± 0.8	n.d.	n.d.	n.d.	n.d.
Sr	7 ± 0.3	600 ± 400	140 ± 0.8	12 ± 8	80 ± 20	70 ± 30	70 ± 50	50 ± 30
Ti^a^	n.d.	800 ± 500	n.d.	61 ± 7	6 ± 4	5 ± 2	5 ± 4	2 ± 1
V	n.d.	40 ± 20	n.d.	60 ± 20	0.3 ± 0.5	0.7 ± 0.6	1.1 ± 0.8	0.2 ± 0.2
Zn*	0.003 ± 0.005	60 ± 20	2.3 ± 0.4	150 ± 50	3600 ± 600	4000 ± 1000	3000 ± 1000	3000 ± 2000

In bold the cases where site A and site B were significantly different (T-test, p = 0.05). Hg and Tl were analyzed and not detected in any specimen.

*n.d*. not detected.

^a^Sediment data were collected as the corresponding oxide.

*Comparable amount was found in the shell control sample.

–Not analyzed.

Aluminum was detected in all tissues, having the highest concentration in the byssus (440 μg g^−1^). Antimuonium was detected in trace in the byssus (4 μg g^−1^) and was absent in all other matrices. Arsenic was not detected in the shell and only traces were detected in the byssus (2 μg g^−1^). A trace of Be was found in soft tissues, but was not detected in the shell and the byssus. Barium, widely present in the sediment (400 μg g^−1^) was observed in traces in the tissues (~ 5 μg g^−1^), especially in the exposed ones. Boron was present in the shell, in a concentration lower than in tissues (~ 20 μg g^−1^) or seawater, but was highly concentrated in the byssus (about 200 μg g^−1^). Cd was not found in seawater and sediment and was present in low concentration in the soft tissues (~ 5 μg g^−1^). The concentration of Cu (80 μg g^−1^) in the byssus it was over 25 times higher than in the sediment and about 5 times higher than in the soft tissues. Iron concentration in the byssus (1100 μg g^−1^) was lower than in the sediment but about 10 times higher than in the soft tissues. Chromium was found in the sediment (26 μg g^−1^) and in trace on the mantle. Traces of Mn were found in shells and byssus (1 μg g^−1^). Mo was widely concentrated in the byssus (20 μg g^−1^), but was almost not detected in the other tissues. Lead was detected in the sediment (36 μg g^−1^) and more concentrated in the byssus (50 μg g^−1^). Tin was present in the sediments (3 μg g^−1^) and detected only in the byssus (1.7 μg g^−1^). Titanium, present in the sediments, was not detected in the shell while it was found concentrated in the byssus (61 μg g^−1^). The concentration of Zn in the environment matrices was lower than that in the byssus (150 μg g^−1^) and tissues. Vanadium was not detected in the shell, but it was concentrated in the byssus (60 μg g^−1^), having almost two times the concentration in the sediment and 60 times the concentration of the highest concentrated tissue. Selenium was detected in low concentration in the byssus (5 μg g^−1^), and the other soft tissues, but only in traces in the shell.

The concentration in the soft tissues provided information on the intake of elements. The gills and the hepatopancreas were used to define whether the element intake preferentially occurred by respiration or ingestion, respectively^51^. The internalization of these elements was defined by evaluating the element content in the muscle tissue, since it is the one less exposed to seawater. The mantle was analyzed for different purposes: (i) as the gills, it exposes a wide surface, thus, it is likely to absorb elements by diffusion; (ii) as the muscle, element can reach this tissues from gills or hepatopancreas only if they have been previously internalized; (iii) it is the tissue mostly related to the shell mineralization, any element found in the shell must also be present in this tissue, otherwise, to come directly from seawater. All this information for each element is summarized in Table 2.

**Table 2:** Summary of the observations on the possible preferential absorption route, eventual internalization, and potential use of external matrices as marker of the metal past-exposure/presence in the organism.

	Environment		Preferentially absorbed by			Internalized	Potential marker		Pollution
	Seawater	Sediment	Diffusion	Respiration	Digestion		Shell	Byssus	
Ag			x					x	
Al		x	x	x		x		x	
As		x			x	x			
B	x	x			x	x	x	x	
Ba	x	x				x	x	x	
Be					x	x			
Cd			x			x			Yes^15,22–24^
Co					x				
Cr		x	x					x	No^21,22^
Cu		x	–	–	–	–		x	No^15,23^
Fe		x	–	–	–	–	x	x	No^24^
Li	x				x	x	x		
Mn	x	x	x	x		x	x	x	Yes^24^
Mo		x	x					x	
Ni		x	–	–	–	–	–	–	
Pb		x	x			x	x	x	Yes^15,22–24^
S	x	x	–	–	–	–	–	–	
Sb								x	
Se		x				x	x	x	
Si		x	–	–	–	–	–	–	
Sn	x	x						x	
Sr		x				x		x	
Ti		x				x		x	
V		x	x					x	
Zn		x				x		x	Yes^24^

When (–) was used the physiological role, or the highly presence in the environment of the element, did not allow an interpretation of the results. Pollution status was defined based on previous data in literature, when possible.

## Discussion

*Pinna nobilis* is a protected species^18^, and currently listed as “Critically Endangered” by the IUCN Red List of Threatened Species (IUCN, 2019) as a consequence of drastic population reduction caused by a mass mortality event^16^. Accordingly, the collection or any human interference with the species will be forbidden as long as the populations is restored. Thus, any adjoin information on this species prior to the massive outbreak is crucial for understanding ecological mechanisms of its relationships with the surrounding environment and its survival. In this view, the possibility of monitoring the animal content of pollutants without vitally damaging it is of primary importance.

To achieve this goal, it has been verified if the analysis of the elements in the acellular tissues (i.e. byssus and shell), which can be sampled without endangering the life of the organism, can provide information on their presence in the environment and in the soft tissues. A follow up, linked to the concentration of the elements in the tissues, is to evaluate the presence of a past pollution by comparison with literature data and to provide an overview on the absorption mode of elements by the organism.

The limited number of specimens (6) can appear as a limit in determining contamination levels of elements in the environment using their concentration in *P. nobilis* tissues. This because the accurate evaluation of concentration and distribution of elements in the tissues, which is influenced both by intrinsic and extrinsic environmental factors^52^, biotic and abiotic factors^53,54^, and sex and spawning conditions^55^, requires a statistically elevated number of samples.

The statistical analysis of element composition of *P. nobilis* growing environment (seawater and sediments) and tissues suggested that all specimens have been exposed to the same environment (unless for Ag and Cr). This observation is also strengthened by the data on element composition in successive annual increments of calcite shell layer indicating that specimens were exposed to similar environments for all life. Unfortunately, an annual record of data on the elemental composition of seawater and sediments in the *P. nobilis* growing sites is not available. The available data regard regions not densely populated by *P. nobilis*, but in proximity of the collection sites. They show the absence of a significant pollution in the seawater and sediments in the last 5 years^56^.

The obtained data (Table 1) have allowed to evaluate the presence of a correlation among elements present in the living environment (sediments and seawater), soft tissues, and external acellular matrices of *P. nobilis*. A correlation among elements in sediments and seawater, and in the byssus and calcite shell layer (acellular matrices) was observed. This occurred for B, Ba, Fe, Mn, Pb and Se. In addition to them, other elements present in the growing environment were detected in the byssus, they were: Ag, Al, Cr, Cu, Mo, Sb, Sn, Sr, Ti, V and Zn. Among these elements Cr, Cu, Fe, Mn, Pb, and Zn may be related to a status of pollution (Table 2)^15,22–24^. In the byssus the concentration of Cr, Sb, Sn, and V was related to the environment and might be used to draw a historical record of the exposure of the organism. This was also observed for B, Ag, As, Mn, Mo, Pb, or Se in relation to soft tissues. All this it proved the initial hypothesis that the element composition of the byssus of *P. nobilis*, can provide information on the growing environment status and of element content in vital soft tissues.

Information on similar correlations are available for other mollusk species (e.g. Ref.^53,57^). A study on the distribution and relationships of Hg, Cd, Pb, Ag, Cu, Zn, Cr, Ni, Co, Mn, and Fe in soft tissue, byssus and shells of *Mytilus edulis trossulus* revealed that the byssus, as compared with the soft tissue, concentrated more effectively Pb, Cu, Cr, and especially Ag, Ni, Mn and Fe, moderately Hg and Zn and less effectively Cd^58^. The capability of the byssus, like soft tissue, selectively and sensitively reflects variations of certain metal concentrations in ambient has been demonstrated for several *Mytilus* and *Mytella* species^59^.

The effects of the presence of no physiological elements on the features of the byssus and the shell of *P. nobilis* can be inferred from knowledge from other mollusks. In a study on *Mytilus californianus* mussel byssus it was demonstrated that the Fe in the DOPA coordination complex can be replaced with V and Al without leading to statistically significant changes in the stiffness and hardness of the material^60^. The presence of no physiological metal ions has been hypothesized to disrupt shell integrity and strength^61^. Alternately, it was suggested that metal ions can modify the activity of proteins during the biomineralization process altering shell microstructure features^62^. A recent study showed that metal ion pollution (zinc, copper and lead) correlated with a significant weakening of shell strength in *Pecten maximus*^63^. These observations have important implications for the adaptability of these biological matrices and for the survival of organisms in a changing habitat exposed to contaminants.

The data on element concentration in the tissues samples can be compared with those from literature for *P. nobilis*^15,22–24^ to address the presence of a past pollution. The concentration of Cu was similar to that reported for organism grew in a not polluted environment^15,23^. Iron concentration was lower than that reported for *P. nobilis* grew in a polluted environment^24^. The concentration of Zn and Mn in soft tissues was comparable or higher than that observed in a *P. nobilis* sample after being transplanted for 3 months in a polluted area^24^, suggesting a light Zn and Mn pollution in the investigated area as occurred. Lead concentration values were between those reported for a clean site and sites with anthropogenic activity^15,23^. Moreover, values 10 time higher in gills and mantle were observed respect to those reported for *P. nobilis* from a pollutes site^22^. Jebali et al.^24^, investigations indicated that our level of Pb in gills was about double the one in a *P. nobilis* after being transplanted for 3 months in a polluted area^24^. On the other hand, in the same comparison, the Pb level in the hepatopancreas appears to be lower. Considering the results of this comparison, a moderate lead pollution was observed in the sites studied, likely due to the harbor proximity. A geographically wide study was performed on Cr contamination in *P. nobilis* tissues^21^, and the comparison of the results suggest that the investigated sites are almost uncontaminated. The content of Cd in the investigated samples is between those reported for *P. nobilis* in a protected area and in two polluted sites^15,23^. In another study the same four tissues considered in presented research were analyzed, showing a short dynamic range between clean and light-polluted areas in *P. nobilis* (ranging between 0.5 to 2 μg g^−1^)^22^. From this knowledge, we could infer that a light Cd pollution had been present in the area of study. This observation is also confirmed from comparison from data from muscle and gills in *P. nobilis* after 3 months from being transplanted in a polluted area^24^.

A discussion on the absorption route of elements among reported Table 2. Silver was detected in a small concentration the muscle or the hepatopancreas, Mo and Sn were absorbed on exposed tissues, Mn was found in tissues in a concentration similar as in the sediment, suggesting for them an uptake by diffusion. As, B, might have been internalized by the ingestion, being accumulated in the tissues. Barium was observed in traces in the tissues, especially in the exposed ones. It was entrapped within the calcite shell layer as already reported in the literature for other species, where is used as a marker for seawater salinity^64^. A concentration similar as that in the exposed tissues was detected in the byssus. Since this concentration was widely lower than in sediment and higher than in seawater, it is probably linked to that accumulated in the tissues of the organism. Traces were found in the byssus, most likely absorbed by seawater. Strontium, as already reported in literature, was found in the shell, where has been used as a proxy of seawater temperature^65^. Se, Ti and Al were found in all the tissues, showing no preferential adsorption route. Zn was probably internalized in the byssus from the organism. Vanadium was mildly internalized and probably was absorbed on exposed tissues by mere diffusion. Byssus is mostly a marker of V. Selenium was not detected in seawater, its presence into the byssus could come from the organism or by slow diffusion from the sediment. Despite the organism was exposed to an undetectable amount or had short time exposures to higher concentrations, Cd was detected in the tissues, but not in shell or byssus. Cd was not incorporated in the shell despite it was largely present in the mantle. A similar concentration in byssus and sediment was observed, meaning it might be excreted abundantly in the byssus by the tissues or later absorbed and equilibrated from surrounding sediments.

It is important to consider that the intake of an element is probably a combination of the different modes discussed. In this study, the one that is most likely to make a major contribution is highlighted.

## Conclusion

This study has shown that the content of elements in the acellular and not-lethal *P. nobilis* byssus and in minor extent calcite shell layer can provide information on the presence of elements in the organism growing environment and soft tissues. The calcite shell layer showed a low utility for the detection of environmental elements. On the other hand, the byssus contained almost every element the organism had been exposed to. The results showed how metals like Sn and V are strongly related to the environment more than the tissues, being able to concentrate by the byssus in an amount higher than in the environment itself. Cr and Sb were detected in the byssus even if tissues had no more traces of their exposure. Other elements have been observed in the byssus and might show a correlation between byssus and soft tissue content, as Ag, As, Mo, Pb or Se.

Finally, it can be stated that this screening gave a wide range of information that could target new studies on: (i) the collection of the external matrices, shell and byssus, as non-lethal sampling technique for this endangered species; (ii) the use of external matrices as proxies for the exposure of this protected organism to pollutant; (iii) the distribution of elements in *P. nobilis* organism. Moreover, this information can be useful in taking actions to mitigate the pollution risks to which this species is subject and to reconstruct the environment in which *P. nobilis* lived.

## Supplementary Information

Supplementary Information.

## Data Availability

The following files are available free of charge.
