# Supplementary material for: A non-lethal method to assess element content in the endangered Pinna nobilis
Source: Sci Rep. 2021 Sep 28;11:19244. doi: 10.1038/s41598-021-98535-2 (PMC8478926; doi:10.1038/s41598-021-98535-2)
Supplement: Supplementary file 1 — Supplementary Information. [file 41598_2021_98535_MOESM1_ESM.docx]

Supporting information

**A non-lethal method to assess element content in the endangered *Pinna nobilis***

*Devis Montroni,^a,#,^* Andrea Simoni,^b^ Viviana Pasquini,^c^ Enrico Dinelli,^d^ Claudio Ciavatta,^b^ Carla Triunfo,^a,f^ Marco Secci,^c^ Claudio Marzadori,^b^ Piero Addis,^c,*^ and Giuseppe Falini^a,e,*^*

^a^ Dipartimento di Chimica “G. Ciamician”, Alma Mater Studiorum − Università di Bologna, via F. Selmi 2, 40126 Bologna, Italy. ^b^DiSTA, Department of Science and Technology of Agriculture and Environment, Alma Mater Studiorum - Università di Bologna, via Fanin 40, 40127 Bologna, Italy.

^c^ Dipartimento di Scienze della Vita e dell’Ambiente, Università di Cagliari, via Fiorelli 1, 09126 Cagliari, Italy. ^d^ Dipartimento di Scienze Biologiche, Geologiche e Ambientali, Alma Mater Studiorum − Università di Bologna, piazza di Porta San Donato 1, 40126 Bologna, Italy. ^e^ Consiglio Nazionale delle Ricerche, Istituto per lo Studio dei Materiali Nanostrutturati (CNR-ISMN), Via P. Gobetti 101, 40129 Bologna, Italy.

Figure S1: Picture of the muscle scars. p. 2

Figure S2: Optical microscopy images of the shell. p. 2

Table S1: Age and length of the *P. nobilis* specimen collected. p. 3

Table S2: Detection limits of the analysis. p. 3

Table S3: Experimental details on the samples. p. 4

Table S4: Average element composition of seawater, sediments and *P. nobilis* acellular tissues from site A and B. p. 5

Table S5: Average element concentration in the calcite shell layer at different ages for specimens collected in the site A. p. 6

Table S6: Average element concentration in the calcite shell layer at different ages for specimens collected in the site B. p. 7

Table S7: Average element composition of soft tissues from specimens collected in the site A and in the site B. p. 8

Table S8: Physiological element concentration. p. 9


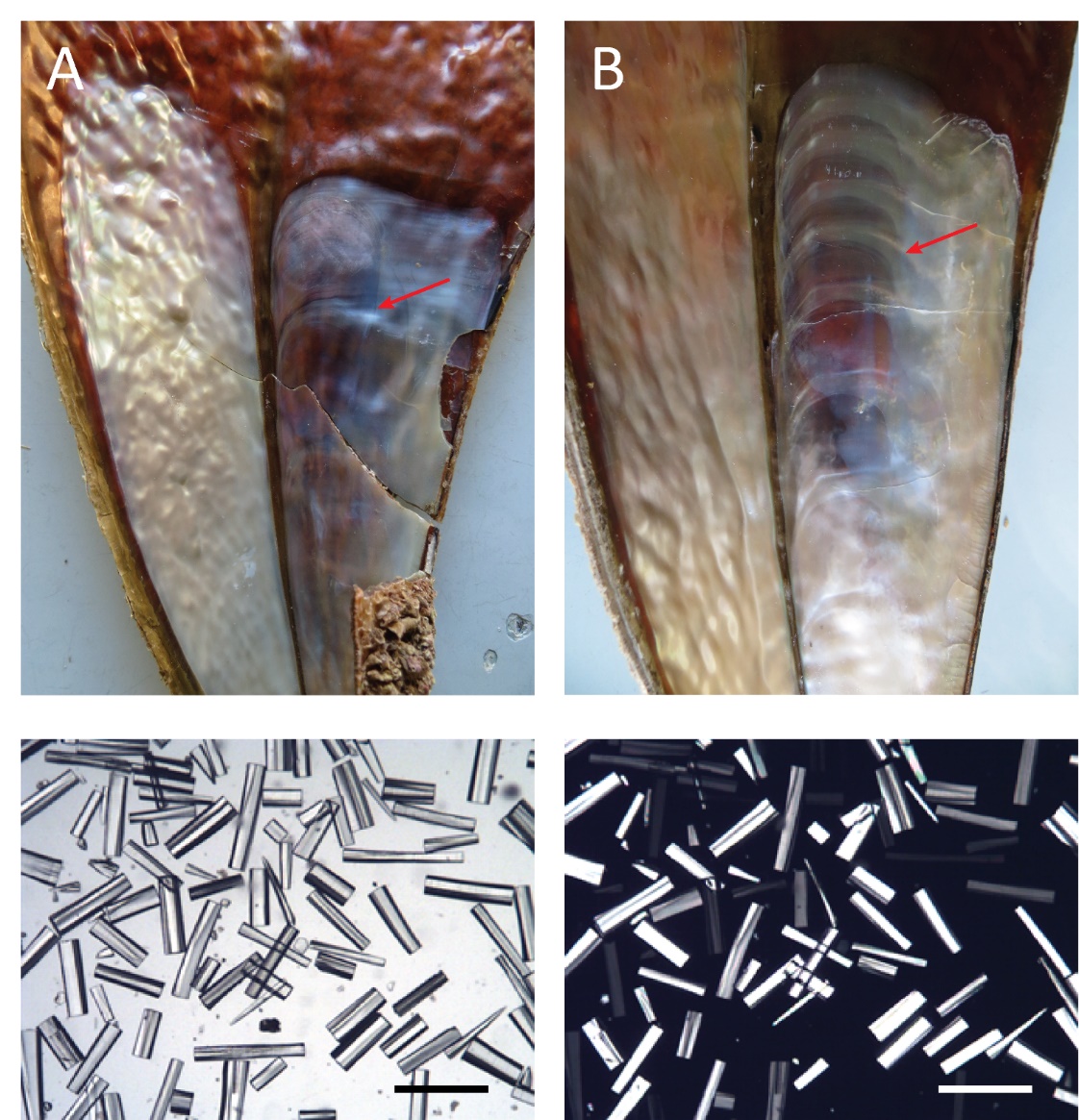


Figure S1: Picture of the muscle scars (red arrow) used to define the age of the samples from the two sites.


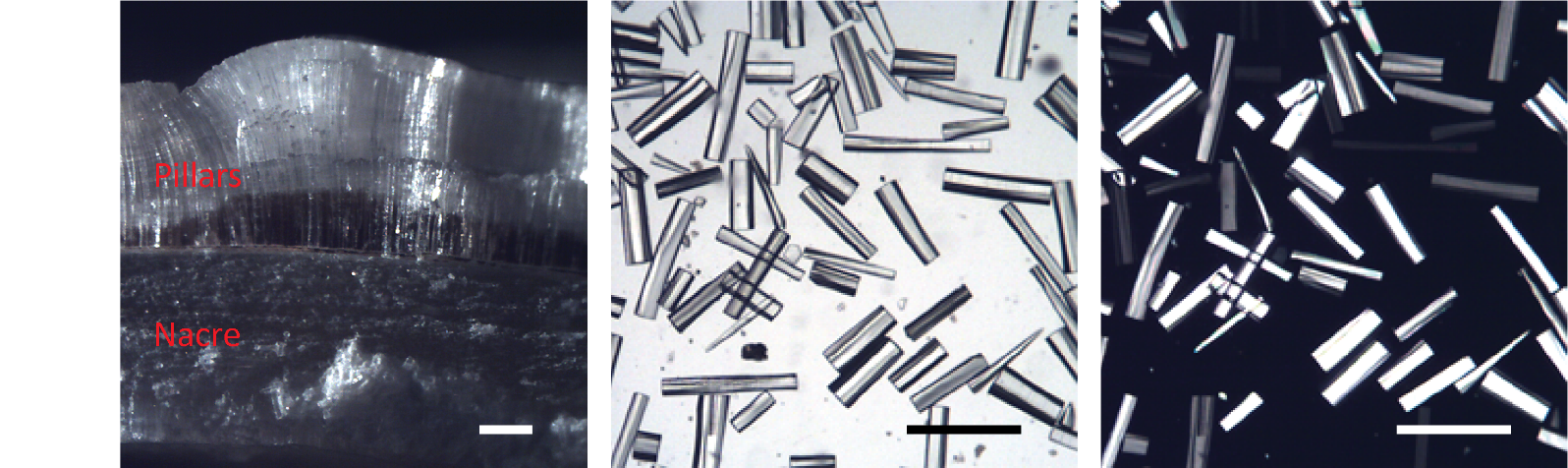


Figure S2: (left) A shell section showing the nacreous and the calcitic layer. Calcite pillars observed using optical microscopy with (right) and without (center) cross-polarizers. Scale bar 300 μm.

Table S1: Age and length of the *P. nobilis* specimen collected.

| **Site** | **Specimen** | **Length** | **Age** |
| --- | --- | --- | --- |
| A | 1 | 32 | 3 |
|  | 2 | 42 | 5 |
| B | 1 | 61 | 10 |
|  | 2 | 62 | 9 |
|  | 3 | 67 | 8 |
|  | 4 | 66 | 7 |

Table S2: Detection limits of the analysis.

|  | **Wavelength** | **Seawater** | **Byssus** | **Shell** | **Tissues** |  |  | **Sediment** |
| --- | --- | --- | --- | --- | --- | --- | --- | --- |
|  | (nm) | (μg·g^-1^) | (μg·g^-1^) | (μg·g^-1^) | (μg·g^-1^) |  |  | (μg·g^-1^) |
| **Ag** | 328.068 | 0.0339 | 0.144 | 0.497 | 0.448 |  | **Ag** | 9 |
| **Al** | 394.401 | 0.145 | 13.3 | 26.8 | 31.2 |  | **Al_2_O_3_** | 0.02 |
| **As** | 189.042 | 0.045 | 6.34 | 88.7 | 7.48 |  | **As** | 2 |
| **B** | 208.959 | 0.021 | 3.64 | 15.4 | 5.42 |  | **B** | 15 |
| **Ba** | 455.404 | 0.005 | 0.138 | 0.434 | 0.214 |  | **Ba** | 5 |
| **Be** | 313.042 | 0.001 | 0.161 | 0.039 | 0.082 |  | **Be** | 1 |
| **Ca** | 183.801 | 0.067 | 24 | 8 | 3 |  | **CaO** | 0.02 |
| **Cd** | 228.802 | 0.009 | 0.203 | 0.152 | 0.093 |  | **Cd** | 0.9 |
| **Co** | 228.616 | 0.0241 | 0.17 | 0.309 | 0.906 |  | **Co** | 2 |
| **Cr** | 267.716 | 0.031 | 0.478 | 0.305 | 0.169 |  | **Cr** | 5 |
| **Cu** | 324.754 | 0.022 | 0.056 | 0.552 | 0.604 |  | **Cu** | 2 |
| **Fe** | 259.941 | 0.015 | 0.255 | 4.09 | 0.43 |  | **Fe_2_O_3_** | 0.02 |
| **Hg** | 184.950 | 0.021 | 0.15 | 3.82 | 1.36 |  | **Hg** | --- |
| **K** | 766.491 | 0.037 | 1 | 7 | 2 |  | **K_2_O** | 0.01 |
| **Li** | 670.780 | 0.002 | 0.086 | 0.295 | 0.164 |  | **Li** | 90 |
| **Mg** | 279.079 | 0.209 | 7 | 42 | 8 |  | **MgO** | 0.02 |
| **Mn** | 257.611 | 0.003 | 0.045 | 0.863 | 0.098 |  | **MnO** | 0.01 |
| **Mo** | 202.095 | 0.024 | 163 | 4.7 | 6.34 |  | **Mo** | 2 |
| **Ni** | 231.604 | 0.0463 | 1.24 | 0.821 | 0.916 |  | **Ni** | 2 |
| **P** | 178.287 | 0.025 | 6 | 6 | 8 |  | **P_2_O_5_** | 0.01 |
| **Pb** | 220.353 | 0.104 | 2270 | 2.9 | 8.34 |  | **Pb** | 3 |
| **S** | 182.034 | 0.025 | 2 | 139 | 9 |  | **S** | 20 |
| **Sb** | 206.833 | 0.098 | 5.9 | 11.4 | 11.4 |  | **Sb** | 4 |
| **Se** | 196.090 | 0.04 | 11.5 | 7.62 | 5.82 |  | **Se** | 4 |
| **Si** | 251.612 | --- | 2 | 1 | 1 |  | **SiO_2_** | 0.02 |
| **Sn** | 189.991 | 0.0193 | 2.82 | 5.62 | 6.36 |  | **Sn** | 2 |
| **Sr** | 407.771 | 0.002 | 0.078 | 0.13 | 0.103 |  | **Sr** | 5 |
| **Ti** | 334.941 | 0.00778 | 5.91 | 3.15 | 10.3 |  | **TiO_2_** | 0.01 |
| **Tl** | 190.864 | 0.171 | 7.93 | 10.9 | 13.8 |  | **Tl** | 8 |
| **V** | 292.464 | 0.06 | 1.14 | 6.42 | 1.95 |  | **V** | 5 |
| **Zn** | 213.856 | 0.006 | 0.44 | 0.517 | 0.164 |  | **Zn** | 5 |

--- Not analyzed

Table S3: Experimental details on the samples. Sample dry weight and volume of dissolution. Due to the dimension of some samples and their conservation we were not able to collect all the tissues.

|  |  | **Byssus** | **Shell** | **Hepatopancreas** | **Gills** | **Mantle** | **Muscle** |
| --- | --- | --- | --- | --- | --- | --- | --- |
| **Weight / g** | A1 | 0.1660 | 1.00602 | 0.021 | 0.212 | 0.244 | 0.208 |
|  | A2 | 0.0307 | 1.00079 | - | 0.201 | 0.265 | - |
|  | B1 | 0.3139 | 1.00574 | 0.0725 | 0.251 | 0.286 | 0.2925 |
|  | B2 | 0.2696 | 1.00573 | - | 0.226 | 0.243 | 0.2518 |
|  | B3 | 0.3396 | 1.02237 | 0.032 | 0.2225 | 0.2594 | 0.2565 |
|  | B4 | 0.4784 | 1.00577 | 0.0443 | 0.224 | 0.257 | 0.245 |
| **Volume /mL** | | 50 | 10 | 20 | 20 | 20 | 20 |

Table S4: Average element composition of seawater, sediments and *P. nobilis* acellular tissues from site A and B. In blue significantly different results between the sites (p = 0.05). The standard deviation is reported.

|  | **Seawater** | | | | | | **Sediment** | | | | | | **Shell** | | | | | | **Byssus** | | | | | |
| --- | --- | --- | --- | --- | --- | --- | --- | --- | --- | --- | --- | --- | --- | --- | --- | --- | --- | --- | --- | --- | --- | --- | --- | --- |
|  | **Site A** | | | **Site B** | | | **Site A** | | | **Site B** | | | **Site A** | | | **Site B** | | | **Site A** | | | **Site B** | | |
|  | (μg·g^-1^) | | | (μg·g^-1^) | | | (μg·g^-1^) | | | (μg·g^-1^) | | | (μg·g^-1^) | | | (μg·g^-1^) | | | (μg·g^-1^) | | | (μg·g^-1^) | | |
| Ag | n.d. | | | n.d. | | | n.d. | | | n.d. | | | n.d. | | | n.d. | | | 0 | ± | 0.04 | 0.25 | ± | 0.09 |
| Al | n.d. | | | n.d. | | | 12,000 | ± | 5,000 | 21,000 | ± | 2,000 | n.d. | | | n.d. | | | 360 | ± | 50 | 480 | ± | 70 |
| As | n.d. | | | n.d. | | | 6 | ± | 2 | 7 | ± | 2 | n.d. | | | n.d. | | | 0 | ± | 2 | 4 | ± | 2 |
| B | 3.7 | ± | 0.1 | 3.6 | ± | 0.1 | 11 ± 15 | | | 9 ± 13 | | | 1.4 | ± | 0.2 | 1.2 | ± | 0.2 | 400 | ± | 400 | 80 | ± | 20 |
| Ba | 0.01 | ± | 0.01 | 0.027 | ± | 0.002 | 200 | ± | 200 | 600 | ± | 40 | 0.9 | ± | 0.04 | 1.3 | ± | 0.1 | 5 | ± | 4 | 4 | ± | 2 |
| Be | n.d. | | | n.d. | | | n.d. | | | n.d. | | | n.d. | | | n.d. | | | n.d. | | | n.d. | | |
| Ca | 330 | ± | 10 | 330 | ± | 10 | 180,000 | ± | 40,000 | 80,000 | ± | 30,000 | 33,900 | ± | 100 | 31,000 | ± | 2,000 | 2,000 | ± | 2,000 | 2,000 | ± | 1,000 |
| Cd | n.d. | | | n.d. | | | n.d. | | | n.d. | | | 0 | ± | 2 | 0.005 | ± | 0.005 | 0 | ± | 0.06 | 0.1 | ± | 0.1 |
| Co | n.d. | | | n.d. | | | 0 | ± | 2 | 1 | ± | 1 | n.d. | | | n.d. | | | 0.2 | ± | 0.2 | 0 | ± | 0.03 |
| Cr | n.d. | | | n.d. | | | 24 | ± | 2 | 28 | ± | 7 | 0.45 | ± | 0.09 | 0.6 | ± | 0.7 | 0 | ± | 0.1 | 1.8 | ± | 0.7 |
| Cu | n.d. | | | n.d. | | | 1 | ± | 1 | 5 | ± | 2 | 0.078 | ± | 0.002 | 0.09 | ± | 0.05 | 50 | ± | 20 | 90 | ± | 40 |
| Fe | n.d. | | | n.d. | | | 5,200 | ± | 400 | 7,000 | ± | 3,000 | 5 | ± | 2 | 9 | ± | 2 | 900 | ± | 100 | 1,200 | ± | 300 |
| K | 426 | ± | 9 | 415 | ± | 4 | 15,000 | ± | 5,000 | 23,000 | ± | 1,000 | 330 | ± | 50 | 210 | ± | 20 | 400 | ± | 400 | 70 | ± | 20 |
| Li | 0.3 | ± | 0.01 | 0.293 | ± | 0.008 | n.d. | | | n.d. | | | 2.71 | ± | 0.06 | 2.4 | ± | 0.1 | 0.3 | ± | 0.3 | 0.2 | ± | 0.07 |
| Mg | 1,280 | ± | 50 | 1,250 | ± | 50 | 21,000 | ± | 8,000 | 10,000 | ± | 5,000 | 2,000 | ± | 100 | 2,000 | ± | 100 | 10 | ± | 10 | 30 | ± | 20 |
| Mn | 0.006 | ± | 0.006 | 0.0125 | ± | 0.0005 | 160 | ± | 30 | 120 | ± | 40 | 1.4 | ± | 0.2 | 1.9 | ± | 0.6 | 0.7 | ± | 0.7 | 1 | ± | 1 |
| Mo | n.d. | | | n.d. | | | 5 | ± | 3 | 4 | ± | 2 | n.d. | | | n.d. | | | 8 | ± | 8 | 30 | ± | 20 |
| Ni | n.d. | | | n.d. | | | 7.5 | ± | 0.5 | 8 | ± | 3 | 0.14 | ± | 0.02 | 0.3 | ± | 0.2 | 10 | ± | 10 | 7 | ± | 1 |
| P | 0.12 | ± | 0.02 | 0.05 | ± | 0.02 | 220 | ± | 40 | 260 | ± | 40 | 1 | ± | 0.1 | 1.1 | ± | 0.7 | n.d. | | | n.d. | | |
| Pb | n.d. | | | n.d. | | | 33 | ± | 3 | 40 | ± | 10 | 2.3 | ± | 0.1 | 2.1 | ± | 0.3 | 30 | ± | 10 | 50 | ± | 10 |
| S | 910 | ± | 30 | 880 | ± | 10 | 5,000 | ± | 3,000 | 5,000 | ± | 3,000 | 2,300 | ± | 100 | 2,200 | ± | 100 | 14,000 | ± | 3,000 | 13,000 | ± | 1,000 |
| Sb | n.d. | | | n.d. | | | n.d. | | | n.d. | | | n.d. | | | n.d. | | | 9 | ± | 9 | 2.1 | ± | 0.6 |
| Se | n.d. | | | n.d. | | | 3 ± 4 | | | 7.1 ± 0.9 | | | 0.06 | ± | 0.06 | 0.1 | ± | 0.01 | 3 | ± | 3 | 6 | ± | 2 |
| Si | n.d. | | | n.d. | | | 150,000 | ± | 80,000 | 30,0000 | ± | 40,000 | 0.2 | ± | 0.1 | 0.4 | ± | 0.2 | 60 | ± | 50 | 30 | ± | 10 |
| Sn | 0.026 | ± | 0.002 | 0.00 | ± | 0.02 | 4 | ± | 4 | 3 | ± | 1 | n.d. | | | n.d. | | | 1 | ± | 1 | 2.1 | ± | 0.2 |
| Sr | 7.1 | ± | 0.3 | 6.9 | ± | 0.2 | 900 | ± | 300 | 310 | ± | 90 | 140.3 | ± | 0.1 | 139.8 | ± | 0.9 | 10 | ± | 10 | 12 | ± | 7 |
| Ti | n.d. | | | n.d. | | | 600 | ± | 600 | 900 | ± | 300 | n.d. | | | n.d. | | | 62 | ± | 3 | 60 | ± | 10 |
| V | n.d. | | | n.d. | | | 50 | ± | 10 | 30 | ± | 20 | n.d. | | | n.d. | | | 40 | ± | 20 | 70 | ± | 10 |
| Zn | 0.006 | ± | 0.006 | 0 | ± | 0.006 | 60 | ± | 10 | 60 | ± | 30 | 2.1 | ± | 0.4 | 2.4 | ± | 0.4 | 130 | ± | 30 | 160 | ± | 60 |

n.d.: Not detected.

Table S5: Average element concentration (μg·g^-1^) in successive annual increments in the calcite shell layer from organisms from site A, according to Table S1. The standard deviation is reported.

| age |  | 1 |  |  |  | 2 |  |  |  | 3 |  |  | 4 |  | 5 |
| --- | --- | --- | --- | --- | --- | --- | --- | --- | --- | --- | --- | --- | --- | --- | --- |
| Ag |  | n.d. |  |  |  | n.d. |  |  |  | n.d. |  |  | n.d. |  | n.d. |
| Al |  | n.d. |  |  |  | n.d. |  |  |  | n.d. |  |  | n.d. |  | n.d. |
| As | 0.24 | ± | 0.06 |  | 0.27 | ± | 0.05 |  | 0.27 | ± | 0.03 |  | 0.28 |  | 0.27 |
| B | 2.5 | ± | 0.9 |  | 5 | ± | 2 |  | 2.7 | ± | 0.6 |  | 2.12 |  | 0.54 |
| Ba | 0.5 | ± | 0.5 |  | 0.18 | ± | 0.00 |  | 0.2 | ± | 0.1 |  | 0.11 |  | 0.28 |
| Be |  | n.d. |  |  |  | n.d. |  |  |  |  |  |  | n.d. |  | n.d. |
| Cd |  | n.d. |  |  |  | n.d. |  |  |  |  |  |  | n.d. |  | n.d. |
| Co |  | n.d. |  |  |  | n.d. |  |  |  |  |  |  | n.d. |  | n.d. |
| Cr |  | n.d. |  |  |  | n.d. |  |  |  |  |  |  | n.d. |  | n.d. |
| Cu |  | n.d. |  |  |  | n.d. |  |  |  |  |  |  | n.d. |  | n.d. |
| Fe | 2 | ± | 2 |  | 4 | ± | 2 |  | 1.4 | ± | 0.6 |  | 1.06 |  | 1.34 |
| Hg |  | n.d. |  |  |  | n.d. |  |  |  | n.d. |  |  | n.d. |  | n.d. |
| K | 80 | ± | 10 |  | 71 | ± | 5 |  | 70 | ± | 20 |  | 61.2 |  | 60.4 |
| Li | 1.4 | ± | 0.1 |  | 1.5 | ± | 0.1 |  | 1.5 | ± | 0.3 |  | 1.4 |  | 1.5 |
| Mg | 800 | ± | 20 |  | 800 | ± | 20 |  | 800 | ± | 20 |  | 804 |  | 800 |
| Mn | 2 | ± | 2 |  | 1.9 | ± | 0.1 |  | 1.0 | ± | 0.5 |  | 0.72 |  | 0.72 |
| Mo | 0.94 | ± | 0.07 |  | 0.95 | ± | 0.04 |  | 0.97 | ± | 0.03 |  | 1.03 |  | 1.02 |
| Na | 2000 | ± | 400 |  | 1860 | ± | 90 |  | 1900 | ± | 400 |  | 1940 |  | 2144 |
| Ni |  | n.d. |  |  |  | n.d. |  |  |  | n.d. |  |  | n.d. |  | n.d. |
| P | 0.5 | ± | 0.2 |  | 0.9 | ± | 0.8 |  | 1.3 | ± | 0.6 |  | n.d. |  | n.d. |
| Pb | 1.2 | ± | 0.4 |  | 1.83 | ± | 0.05 |  | 1.8 | ± | 0.4 |  | 1.81 |  | 1.91 |
| S | 4200 | ± | 200 |  | 4300 | ± | 300 |  | 4300 | ± | 700 |  | 4960 |  | 4680 |
| Sb | 0.27 | ± | 0.02 |  | 0.30 | ± | 0.09 |  | 0.30 | ± | 0.06 |  | 0.30 |  | 0.30 |
| Se | 0.9 | ± | 0.1 |  | 0.80 | ± | 0.04 |  | 0.9 | ± | 0.1 |  | 0.91 |  | 0.98 |
| Si | 17.4 | ± | 14.1 |  | 15.2 | ± | 6.2 |  | 23.9 | ± | 4.1 |  | 17.9 |  | 16.6 |
| Sn |  | n.d. |  |  |  | n.d. |  |  |  | n.d. |  |  | n.d. |  | n.d. |
| Sr | 110.2 | ± | 0.1 |  | 110.2 | ± | 0.1 |  | 111 | ± | 1 |  | 110.1 |  | 110.1 |
| Ti | 0.8 | ± | 0.0 |  | 2 | ± | 1 |  | 0.8 | ± | 0.2 |  | 0.9 |  | 1.9 |
| Tl |  | n.d. |  |  |  | n.d. |  |  |  | n.d. |  |  | n.d. |  | n.d. |
| V |  | n.d. |  |  |  | n.d. |  |  |  | n.d. |  |  | n.d. |  | n.d. |
| Zn | 1.0 | ± | 0.3 |  | 1.5 | ± | 0.4 |  | 0.72 | ± | 0.02 |  | 0.7 |  | 0.6 |

n.d.: Not detected. * Only one sample was available.

Table S6: Average element concentration (μg·g^-1^) in successive annual increments in the calcite shell layer from organisms from site B, according to Table S1. The standard deviation is reported.

| age |  | 1 |  |  |  | 2 |  |  |  | 3 |  |  |  | 4 |  |  |  | 5 |  |  |  | 6 |  |  |  | 7 |  |  |  | 8 |  |  | 9* |  | 10* |
| --- | --- | --- | --- | --- | --- | --- | --- | --- | --- | --- | --- | --- | --- | --- | --- | --- | --- | --- | --- | --- | --- | --- | --- | --- | --- | --- | --- | --- | --- | --- | --- | --- | --- | --- | --- |
| Ag |  | n.d. |  |  |  | n.d. |  |  |  | n.d. |  |  |  | n.d. |  |  |  | n.d. |  |  |  | n.d. |  |  |  | n.d. |  |  |  | n.d. |  |  | n.d. |  | n.d. |
| Al |  | n.d. |  |  |  | n.d. |  |  |  | n.d. |  |  |  | n.d. |  |  |  | n.d. |  |  |  | n.d. |  |  |  | n.d. |  |  |  | n.d. |  |  | n.d. |  | n.d. |
| As | 0.22 | ± | 0.01 |  | 0.23 | ± | 0.07 |  | 0.30 | ± | 0.04 |  | 0.27 | ± | 0.00 |  | 0.26 | ± | 0.04 |  | 0.34 | ± | 0.02 |  | 0.26 | ± | 0.04 |  | 0.27 | ± | 0.01 |  | 0.27 |  | 0.26 |
| B | 6.66 | ± | 0.03 |  | 3 | ± | 1 |  | 2 | ± | 1 |  | 2.85 | ± | 0.02 |  | 3.0 | ± | 0.9 |  | 3 | ± | 2 |  | 2.2 | ± | 0.6 |  | 2.86 | ± | 1.74 |  | 1.36 |  | 1.87 |
| Ba | 0.12 | ± | 0.00 |  | 0.07 | ± | 0.06 |  | 0.04 | ± | 0.02 |  | 0.08 | ± | 0.00 |  | 0.08 | ± | 0.03 |  | 0.10 | ± | 0.05 |  | 0.17 | ± | 0.04 |  | 0.4 | ± | 0.4 |  | 0.09 |  | 0.60 |
| Be |  | n.d. |  |  |  | n.d. |  |  |  | n.d. |  |  |  | n.d. |  |  |  | n.d. |  |  |  | n.d. |  |  |  | n.d. |  |  |  | n.d. |  |  | n.d. |  | n.d. |
| Cd |  | n.d. |  |  |  | n.d. |  |  |  | n.d. |  |  |  | n.d. |  |  |  | n.d. |  |  |  | n.d. |  |  |  | n.d. |  |  |  | n.d. |  |  | n.d. |  | n.d. |
| Co |  | n.d. |  |  |  | n.d. |  |  |  | n.d. |  |  |  | n.d. |  |  |  | n.d. |  |  |  | n.d. |  |  |  | n.d. |  |  |  | n.d. |  |  | n.d. |  | n.d. |
| Cr |  | n.d. |  |  |  | n.d. |  |  |  | n.d. |  |  |  | n.d. |  |  |  | n.d. |  |  |  | n.d. |  |  |  | n.d. |  |  |  | n.d. |  |  | n.d. |  | n.d. |
| Cu |  | n.d. |  |  |  | n.d. |  |  |  | n.d. |  |  |  | n.d. |  |  |  | n.d. |  |  |  | n.d. |  |  |  | n.d. |  |  |  | n.d. |  |  | n.d. |  | n.d. |
| Fe | 2.07 | ± | 0.03 |  | 2 | ± | 1 |  | 0.7 | ± | 0.1 |  | 1.07 | ± | 0.01 |  | 1.3 | ± | 0.3 |  | 1.4 | ± | 0.6 |  | 1.5 | ± | 0.5 |  | 1.5 | ± | 0.6 |  | 0.98 |  | 1.94 |
| Hg |  | n.d. |  |  |  | n.d. |  |  |  | n.d. |  |  |  | n.d. |  |  |  | n.d. |  |  |  | n.d. |  |  |  | n.d. |  |  |  | n.d. |  |  | n.d. |  | n.d. |
| K | 64.6 | ± | 0.2 |  | 51 | ± | 5 |  | 51 | ± | 3 |  | 51.8 | ± | 0.1 |  | 49 | ± | 4 |  | 49 | ± | 2 |  | 50 | ± | 1 |  | 52.0 | ± | 0.8 |  | 48.8 |  | 53.6 |
| Li | 1.4 | ± | 0.0 |  | 1.3 | ± | 0.1 |  | 1.3 | ± | 0.1 |  | 1.3 | ± | 0.0 |  | 1.2 | ± | 0.1 |  | 1.2 | ± | 0.1 |  | 1.3 | ± | 0.1 |  | 1.4 | ± | 0.0 |  | 1.3 |  | 1.5 |
| Mg | 802 | ± | 0 |  | 801 | ± | 2 |  | 800 | ± | 0 |  | 801 | ± | 0 |  | 801 | ± | 2 |  | 800 | ± | 0 |  | 801 | ± | 2 |  | 801 | ± | 2 |  | 800 |  | 804 |
| Mn | 1.19 | ± | 0.01 |  | 1.0 | ± | 0.3 |  | 0.8 | ± | 0.3 |  | 0.98 | ± | 0.01 |  | 0.8 | ± | 0.4 |  | 0.8 | ± | 0.4 |  | 0.9 | ± | 0.4 |  | 0.8 | ± | 0.6 |  | 0.49 |  | 0.77 |
| Mo | 0.96 | ± | 0.00 |  | 0.93 | ± | 0.06 |  | 0.95 | ± | 0.07 |  | 0.96 | ± | 0.00 |  | 0.92 | ± | 0.09 |  | 0.93 | ± | 0.06 |  | 0.94 | ± | 0.06 |  | 0.96 | ± | 0.05 |  | 0.97 |  | 1.02 |
| Na | 1950 | ± | 5 |  | 1600 | ± | 200 |  | 1600 | ± | 100 |  | 1774 | ± | 4 |  | 1600 | ± | 200 |  | 1600 | ± | 100 |  | 1800 | ± | 100 |  | 2000 | ± | 100 |  | 1748 |  | 2100 |
| Ni |  | n.d. |  |  |  | n.d. |  |  |  | n.d. |  |  |  | n.d. |  |  |  | n.d. |  |  |  | n.d. |  |  |  | n.d. |  |  |  | n.d. |  |  | n.d. |  | n.d. |
| P | 0.70 | ± | 0.01 |  | 0.5 | ± | 0.2 |  | 1 | ± | 1 |  | 0.84 | ± | 0.01 |  | 0.6 | ± | 0.5 |  | 1.0 | ± | 0.2 |  | 0.9 | ± | 0.8 |  | 0.6 | ± | 0.4 |  | 0.54 |  | 0.97 |
| Pb | 1.99 | ± | 0.02 |  | 1.2 | ± | 0.7 |  | 1.1 | ± | 0.6 |  | 1.50 | ± | 0.01 |  | 1.4 | ± | 0.3 |  | 1.37 | ± | 0.06 |  | 1.4 | ± | 0.4 |  | 1.5 | ± | 0.3 |  | 1.21 |  | 1.04 |
| S | 4290 | ± | 10 |  | 3700 | ± | 400 |  | 3800 | ± | 300 |  | 4169 | ± | 8 |  | 3800 | ± | 600 |  | 3900 | ± | 200 |  | 4000 | ± | 200 |  | 4400 | ± | 200 |  | 4000 |  | 4720 |
| Sb | 0.28 | ± | 0.00 |  | 0.28 | ± | 0.04 |  | 0.31 | ± | 0.09 |  | 0.32 | ± | 0.00 |  | 0.29 | ± | 0.05 |  | 0.38 | ± | 0.07 |  | 0.38 | ± | 0.06 |  | 0.38 | ± | 0.05 |  | 0.32 |  | 0.32 |
| Se | 0.93 | ± | 0.01 |  | 1.4 | ± | 0.5 |  | 1.9 | ± | 0.4 |  | 2.22 | ± | 0.01 |  | 1.8 | ± | 0.6 |  | 1.5 | ± | 0.4 |  | 1.3 | ± | 0.5 |  | 1.2 | ± | 0.3 |  | 1.08 |  | 1.01 |
| Si | 20.9 | ± | 0.2 |  | 12 | ± | 3 |  | 18 | ± | 7 |  | 19.2 | ± | 0.2 |  | 11 | ± | 4 |  | 13 | ± | 1 |  | 14 | ± | 2 |  | 16 | ± | 3 |  | 13.8 |  | 24.1 |
| Sn | n.d. | ± | n.d. |  | n.d. | ± | n.d. |  | n.d. | ± | n.d. |  | n.d. | ± | n.d. |  | n.d. | ± | n.d. |  | n.d. | ± | n.d. |  | n.d. | ± | n.d. |  | n.d. | ± | n.d. |  | n.d. |  | n.d. |
| Sr | 111.3 | ± | 0.1 |  | 112.8 | ± | 2.0 |  | 112.1 | ± | 2.0 |  | 111.3 | ± | 0.0 |  | 112.2 | ± | 1.9 |  | 112.5 | ± | 1.0 |  | 112.6 | ± | 1.7 |  | 110.5 | ± | 0.3 |  | 113.2 |  | 110.2 |
| Ti | 0.8 | ± | 0.0 |  | 0.7 | ± | 0.1 |  | 0.7 | ± | 0.1 |  | 0.7 | ± | 0.0 |  | 0.7 | ± | 0.1 |  | 1 | ± | 2 |  | 0.7 | ± | 0.1 |  | 1.0 | ± | 0.3 |  | 0.7 |  | 0.8 |
| Tl |  | n.d. |  |  |  | n.d. |  |  |  | n.d. |  |  |  | n.d. |  |  |  | n.d. |  |  |  | n.d. |  |  |  | n.d. |  |  |  | n.d. |  |  | n.d. |  | n.d. |
| V |  | n.d. |  |  |  | n.d. |  |  |  | n.d. |  |  |  | n.d. |  |  |  | n.d. |  |  |  | n.d. |  |  |  | n.d. |  |  |  | n.d. |  |  | n.d. |  | n.d. |
| Zn | 1.0 | ± | 0.0 |  | 0.7 | ± | 0.2 |  | 0.5 | ± | 0.1 |  | 0.8 | ± | 0.0 |  | 0.4 | ± | 0.1 |  | 0.4 | ± | 0.1 |  | 0.5 | ± | 0.2 |  | 0.3 | ± | 0.0 |  | 0.2 |  | 0.3 |

n.d.: Not detected. * Only one sample was available.

Table S7: Average element composition of soft tissues from specimens collected in the site A and in the site B. The standard deviation is reported.

|  | **Hepatopancreas** | | | | | | **Gills** | | | | | | **Mantle** | | | | | | **Muscle** | | | | | |
| --- | --- | --- | --- | --- | --- | --- | --- | --- | --- | --- | --- | --- | --- | --- | --- | --- | --- | --- | --- | --- | --- | --- | --- | --- |
|  | **Site A** | | | **Site B** | | | **Site A** | | | **Site B** | | | **Site A** | | | **Site B** | | | **Site A** | | | **Site B** | | |
|  | (μg·g^-1^) | | | (μg·g^-1^) | | | (μg·g^-1^) | | | (μg·g^-1^) | | | (μg·g^-1^) | | | (μg·g^-1^) | | | (μg·g^-1^) | | | (μg·g^-1^) | | |
| Ag | 0 | ± | 0.07 | 0.3 | ± | 0.2 | 2.32 | ± | 0.04 | 0.49 | ± | 0.05 | 1.7 | ± | 0.4 | 1.2 | ± | 0.6 | 0.4 | ± | 0.2 | 0.4 | ± | 0.2 |
| Al | 50 | ± | 80 | 60 | ± | 70 | 37 | ± | 6 | 100 | ± | 100 | 300 | ± | 300 | 110 | ± | 40 | 0 | ± | 9 | 23 | ± | 9 |
| As | 40 | ± | 20 | 50 | ± | 20 | 40 | ± | 20 | 22 | ± | 8 | 40 | ± | 30 | 90 | ± | 20 | 18 | ± | 6 | 19 | ± | 6 |
| B | 40 | ± | 10 | 30 | ± | 10 | 25 | ± | 7 | 12 | ± | 6 | 20 | ± | 10 | 20 | ± | 2 | 17 | ± | 3 | 17 | ± | 3 |
| Ba | 6 | ± | 1 | 5 | ± | 1 | 1.65 | ± | 0.03 | 7 | ± | 8 | 3 | ± | 2 | 3 | ± | 1 | 0.8 | ± | 0.2 | 1.3 | ± | 0.2 |
| Be | 0.32 | ± | 0.06 | 0.16 | ± | 0.06 | 0.033 | ± | 0.004 | 0.05 | ± | 0.02 | 0.03 | ± | 0.01 | 0.053 | ± | 0.004 | 0.023 | ± | 0.004 | 0.023 | ± | 0.004 |
| Ca | 4,000 | ± | 1,000 | 3,000 | ± | 1,000 | 3,100 | ± | 100 | 3,000 | ± | 1,000 | 2,000 | ± | 1,000 | 10,000 | ± | 20,000 | 1,700 | ± | 900 | 2,200 | ± | 900 |
| Cd | 10.3 | ± | 0.3 | 1 | ± | 1 | 14 | ± | 3 | 4 | ± | 2 | 20 | ± | 10 | 2 | ± | 1 | 8 | ± | 2 | 2 | ± | 2 |
| Co | 74 | ± | 1 | 5 | ± | 1 | 3 | ± | 3 | 0.3 | ± | 0.4 | 0.8 | ± | 0.8 | 0.1 | ± | 0.2 | 0.5 | ± | 0.5 | 0.1 | ± | 0.1 |
| Cr | n.d. | | | n.d. | | | 0.03 | ± | 0.03 | 0.1 | ± | 0.2 | 0.4 | ± | 0.4 | 0.3 | ± | 0.1 | n.d. | | | n.d. | | |
| Cu | 32 | ± | 7 | 15 | ± | 7 | 30 | ± | 10 | 7 | ± | 2 | 18 | ± | 7 | 8 | ± | 2 | 8 | ± | 2 | 4 | ± | 2 |
| Fe | 450 | ± | 30 | 290 | ± | 30 | 210 | ± | 90 | 200 | ± | 100 | 300 | ± | 300 | 130 | ± | 20 | 40 | ± | 30 | 70 | ± | 30 |
| K | 15,000 | ± | 2,000 | 16,000 | ± | 2,000 | 6,000 | ± | 3,000 | 2,700 | ± | 800 | 4,000 | ± | 3,000 | 6,000 | ± | 1,000 | 5,300 | ± | 400 | 5,300 | ± | 400 |
| Li | 1.5 | ± | 0.7 | 1.7 | ± | 0.7 | 0.7 | ± | 0.2 | 0.5 | ± | 0.4 | 0.9 | ± | 0.9 | 1.1 | ± | 0.2 | 0.6 | ± | 0.08 | 0.77 | ± | 0.08 |
| Mg | 8,000 | ± | 2,000 | 7,000 | ± | 2,000 | 4,000 | ± | 800 | 3,000 | ± | 1,000 | 3,000 | ± | 2,000 | 4,600 | ± | 700 | 3,100 | ± | 300 | 3,700 | ± | 300 |
| Mn | 530 | ± | 40 | 210 | ± | 40 | 500 | ± | 90 | 400 | ± | 300 | 300 | ± | 200 | 170 | ± | 40 | 220 | ± | 70 | 110 | ± | 70 |
| Mo | n.d. | | | n.d. | | | 1.2 | ± | 0.3 | 0 | ± | 0.6 | 0.8 | ± | 0.1 | 0 | ± | 0.5 | n.d. | | | n.d. | | |
| Ni | 6 | ± | 1 | 2 | ± | 1 | 5.6 | ± | 0.8 | 1.8 | ± | 0.9 | 4 | ± | 3 | 1 | ± | 0.3 | 2 | ± | 1 | 1 | ± | 1 |
| P | 4,600 | ± | 700 | 4,500 | ± | 700 | 5,500 | ± | 300 | 5,000 | ± | 1,000 | 3,300 | ± | 300 | 3,200 | ± | 300 | 2,000 | ± | 2,000 | 3,000 | ± | 2,000 |
| Pb | 12 | ± | 6 | 6 | ± | 6 | 13 | ± | 3 | 12 | ± | 8 | 12 | ± | 8 | 13 | ± | 3 | 1 | ± | 7 | 7 | ± | 7 |
| S | 19,000 | ± | 2,000 | 17,000 | ± | 2,000 | 13,000 | ± | 4,000 | 7,400 | ± | 800 | 9,000 | ± | 4,000 | 11,000 | ± | 1,000 | 9,500 | ± | 600 | 9,100 | ± | 600 |
| Sb | n.d. | | | n.d. | | | n.d. | | | n.d. | | | n.d. | | | n.d. | | | n.d. | | | n.d. | | |
| Se | 12.19 | ± | 0.07 | 7.64 | ± | 0.07 | 9 | ± | 3 | 5 | ± | 1 | 6 | ± | 3 | 8 | ± | 2 | 2.5 | ± | 0.7 | 2.6 | ± | 0.7 |
| Si | 50 | ± | 20 | 60 | ± | 20 | 16.9 | ± | 0.6 | 19 | ± | 6 | 26 | ± | 2 | 21 | ± | 7 | 6 | ± | 4 | 16 | ± | 4 |
| Sn | 9 | ± | 2 | 2 | ± | 2 | 0 | ± | 0.4 | 0.2 | ± | 0.3 | n.d. | | | n.d. | | | n.d. | | | n.d. | | |
| Sr | 100 | ± | 20 | 70 | ± | 20 | 71 | ± | 7 | 70 | ± | 40 | 50 | ± | 30 | 80 | ± | 60 | 30 | ± | 30 | 60 | ± | 30 |
| Ti | 0 | ± | 1 | 8 | ± | 1 | 4.45 | ± | 0.03 | 5 | ± | 3 | 9 | ± | 7 | 3.5 | ± | 0.4 | 2 | ± | 1 | 3 | ± | 1 |
| V | n.d. | | | 0.4 | ± | 0.6 | 1.2 | ± | 0.7 | 0.6 | ± | 0.5 | 2 | ± | 2 | 0.8 | ± | 0.3 | 0.3 | ± | 0.1 | 0.2 | ± | 0.2 |
| Zn | 2,600 | ± | 400 | 3,900 | ± | 400 | 3,000 | ± | 2,000 | 4,300 | ± | 400 | 2,000 | ± | 800 | 3,900 | ± | 700 | 1,000 | ± | 2,000 | 3,000 | ± | 2,000 |

n.d.: Not detected.

Table S8: Average concentration of physiological elements. Average element concentration in the specimen collected (A and B considered as a unique set). In light blue the cases where site A and site B were significantly different (T-test, p = 0.05). The standard deviation is reported.

|  | **Seawater** | | | | | **Sediment** | | | | | | **Shell** | | | | | **Byssus** | | | | |
| --- | --- | --- | --- | --- | --- | --- | --- | --- | --- | --- | --- | --- | --- | --- | --- | --- | --- | --- | --- | --- | --- |
|  | (μg·g^-1^) | | | | | (μg·g^-1^) | | | | | | (μg·g^-1^) | | | | | (μg·g^-1^) | | | | |
| Ca | 330 | ± | | 10 | | 130,000 | | | ± | | 60,000 | 32,000 | | ± | | 2,000 | 2,000 | | ± | | 1,000 |
| K | 421 | ± | | 9 | | 19,000 | | | ± | | 5,000 | 250 | | ± | | 60 | 200 | | ± | | 200 |
| Mg | 1,260 | ± | | 50 | | 16,000 | | | ± | | 9,000 | 2,000 | | ± | | 100 | 30 | | ± | | 20 |
| P | 0,08 | ± | | 0,04 | | 240 | | | ± | | 40 | 1,1 | | ± | | 0,6 | n.d. | | | | |
|  |  |  | |  | |  | | |  | |  |  | |  | |  |  | | | | |
|  | **Hepatopancreas** | | | | | | **Gills** | | | | | **Mantle** | | | | | **Muscle** | | | | |
|  | (μg·g^-1^) | | | | | | (μg·g^-1^) | | | | | (μg·g^-1^) | | | | | (μg·g^-1^) | | | | |
| Ca | 3,600 | | ± | | 900 | | 3,000 | ± | | 1,000 | | 10,000 | ± | | 20,000 | | 2,100 | ± | | 800 | |
| K | 16,000 | | ± | | 1,000 | | 4,000 | ± | | 2,000 | | 6,000 | ± | | 2,000 | | 5,300 | ± | | 300 | |
| Mg | 7,000 | | ± | | 1,000 | | 3,000 | ± | | 1,000 | | 4,000 | ± | | 1,000 | | 3,700 | ± | | 300 | |
| P | 4,500 | | ± | | 500 | | 5,200 | ± | | 800 | | 3,200 | ± | | 300 | | 3,000 | ± | | 1,000 | |

n.d.: Not detected.
